# A Case of Ascending Colon Cancer Resected by Laparoscopic Right Hemicolectomy with Intracorporeal Anastomosis after Total Gastrectomy: A Case Report

**DOI:** 10.70352/scrj.cr.25-0370

**Published:** 2025-11-13

**Authors:** Atomu Suzuki, Shin Yoshida, Tsunenori Yamamoto, Masanori Murakami, Yukiko Nagashima, Kazuhiko Sakamoto, Noboru Yahara, Shigefumi Yoshino

**Affiliations:** Department of Surgery, NHO Kanmon Medical Center, Shimonoseki, Yamaguchi, Japan

**Keywords:** after total gastrectomy, intracorporeal anastomosis, right colectomy

## Abstract

**INTRODUCTION:**

There are few reports of treatment strategies for ascending colon cancer after total gastrectomy. We report a case of intracorporeal anastomosis was performed for ascending colon cancer after total gastrectomy with Roux-en-Y reconstruction.

**CASE PRESENTATION:**

A 70-year-old man was referred to our institution due to a primary complaint of blood stool. A colonoscopy showed a Type 2 tumor near the hepatic fold of the ascending colon. The clinical diagnosis was ascending colon cancer. He had a history of open total gastrectomy (Roux-en-Y, retrocolic route) and cholecystectomy for gastric cancer in his 40s. Laparoscopic right hemicolectomy with intracorporeal anastomosis was performed. To perform an extracorporeal anastomosis, it was necessary to release adhesions between the reconstructed jejunum and the left-sided transverse colon and mobilize the splenic flexure. If the reconstructed jejunum was damaged, there will be a possibility of redoing the esophago-jejunostomy. By performing an intracorporeal anastomosis, surgery was accomplished with minimal mobilization and without requiring adhesion release between the reconstructed jejunum and the transverse colon. The patient’s postoperative course was uneventful, and he was discharged at 8 days postoperatively.

**CONCLUSIONS:**

Intracorporeal anastomosis may represent a useful and safe option when performing laparoscopic right colectomy in patients with a history of total gastrectomy.

## INTRODUCTION

Intracorporeal anastomosis has been reported to offer several advantages over extracorporeal anastomosis, including reduced postoperative pain, earlier recovery of bowel function, lower incidence of postoperative complications, and shorter hospital stays.^1)^ Furthermore, intracorporeal anastomosis is considered to reduce the need for bowel mobilization and dissection required to bring the bowel outside the abdominal cavity, thereby minimizing the risk of bleeding associated with mesenteric traction during bowel resection and anastomosis.^2)^ In patients undergoing right hemicolectomy after total gastrectomy, extensive adhesions in the upper abdomen are anticipated. During dissection, injury to the elevated jejunal mesentery may occur, potentially necessitating re-anastomosis of the esophagojejunostomy. There are very few reported cases of laparoscopic right colectomy with intracorporeal anastomosis in patients after total gastrectomy.

We experienced a case in which intracorporeal anastomosis was safely and effectively performed during laparoscopic right hemicolectomy following total gastrectomy with Roux-en-Y reconstruction. Given the limited number of similar reports, we present this case.

## CASE PRESENTATION

**Fig. 1:**
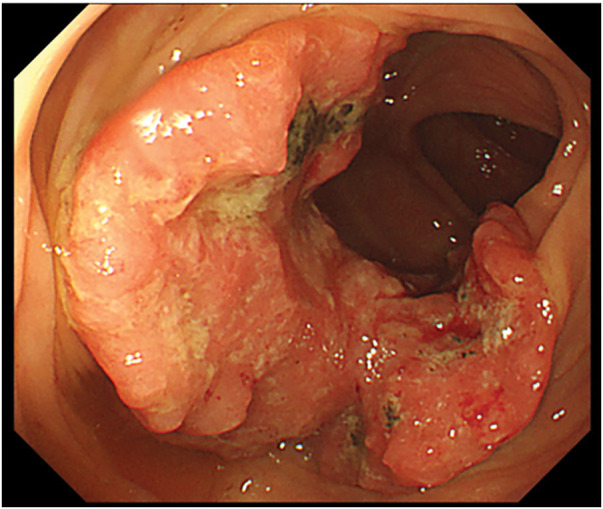
Colonoscopy showed a 1/2 circumferential Type 2 tumor near the hepatic fold in the ascending colon.

**Fig. 2:**
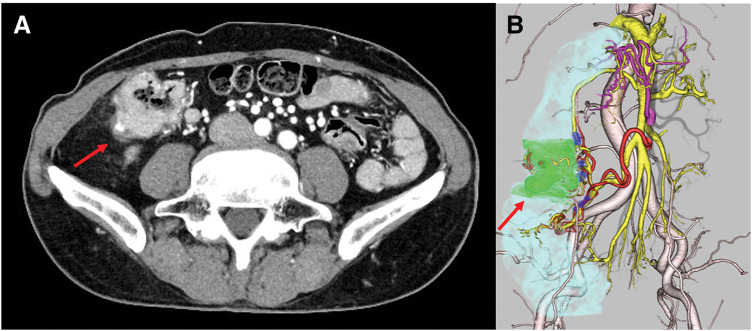
Findings of contrast-enhanced abdominal CT (**A**) and 3D reconstructed CT (**B**). Contrast-enhanced abdominal CT and 3D reconstructed CT revealed an enhanced mass in the ascending colon with a fat stranding sign near the hepatic fold (red arrow).

**Fig. 3:**
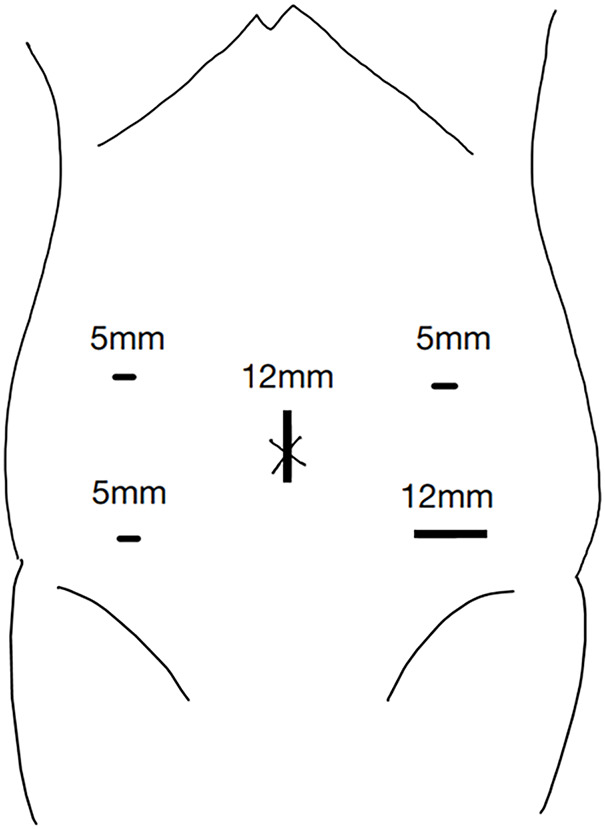
Trocar placement. We performed laparoscopic surgery using 5 ports.

**Fig. 4:**
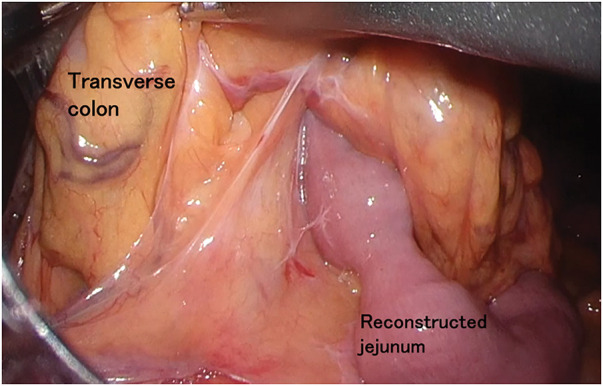
Intraoperative photography: representation of the previous reconstruction. The elevated jejunum is brought up through a retrocolic route for Roux-en-Y reconstruction following total gastrectomy.

**Fig. 5:**
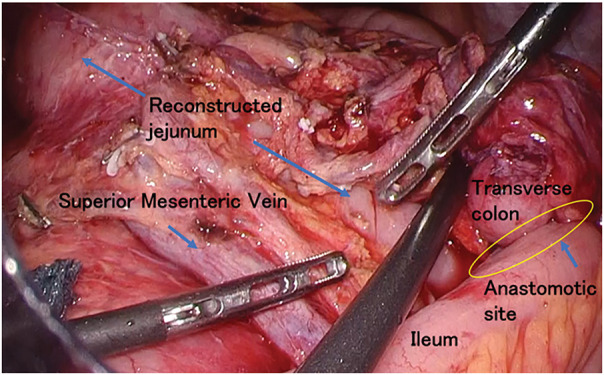
Intraoperative view after right hemicolectomy and reconstruction with intracorporeal anastomosis. We performed a laparoscopic right hemicolectomy with D3 lymph node dissection, and an intracorporeal overlap anastomosis (yellow circle) between the ileum and the transverse colon near the ventral side of the reconstructed jejunum (blue allow).

A 70-year-old man visited our institution for bloody stools. He had a medical history of open total gastrectomy with retro-colic Roux-en-Y reconstruction for gastric cancer in his 40s. He also underwent a cholecystectomy at the same time. Colonoscopy showed a 1/2 circumferential Type 2 tumor near the hepatic fold in the ascending colon (**Fig. 1**). Biopsy findings from the primary tumor revealed a Group 5, moderately-differentiated adenocarcinoma. Laboratory data revealed mild anemia with Hb 9.3 g/dL. The tumor marker levels were as follows: carcinoembryonic antigen, 3.9 ng/dL (upper reference limit, 5.0 ng/dL); carbohydrate antigen 19-9, 4.1 U/mL (upper reference limit, 37.0 U/mL), within the standard range. There were no other abnormal findings of note. Contrast-enhanced CT revealed an enhanced mass in the ascending colon with a fat stranding sign near the hepatic fold. Two enlarged lymph nodes were observed near the tumor in the ascending colon. No distant metastasis was observed (**Fig. 2**). The diagnosis was ascending colon cancer, and the clinical stage was cT4aN1bM0, cStageIIIb (UICC 9th edition). Based on the above preoperative diagnosis, we planned to perform a laparoscopic right hemicolectomy with D3 lymph node dissection. Five ports were placed (**Fig. 3**). Extensive adhesions were found in the abdominal cavity, and the transverse colonic mesentery was adhered to the abdominal wall, so the adhesions were carefully dissected sharply and bluntly. The tumor was located near the hepatic fold of the ascending colon. Retroperitoneal dissection was preceded. The transverse mesocolon was elevated cranially, and the lifted jejunum with the retro-colic route was identified (**Fig. 4**). The ileocolic artery and ileocolic vein were each dissected at their roots, and lymph node dissection was performed along the left margin of the superior mesenteric vein. The right colonic artery was absent, and the accessory right colic vein was transected. The right branch of the middle colonic artery and the middle colonic vein were dissected at the root. We transitioned to a head-side approach. The transverse colon was expanded to the foot side. The mesenteric attachment of the transverse colon was dissected at the inferior border of the pancreas. The outer part of the ascending colon was dissected from the ileum, and the hepatic fold was dissected, completing the mobilization. The planned line of dissection of the colon on the anorectal side was approximately 10 cm anorectally from the tumor. It became the right side of the ventral side of the reconstructed jejunum. The mesentery of the small intestine and transverse colon were each dissected; the ileum was dissected with a Powered Echelon Vlex (Ethicon, Somerville, NJ, USA) 60-mm blue cartridge and the transverse colon with a 60-mm gold cartridge. Before anastomosis, 12.5 mg of indocyanine green (ICG) was injected intravenously to check the blood flow in the ileum and transverse colon. Fluorescence was observed 31 seconds after injection, confirming adequate intestinal perfusion. Reconstruction was performed with an overlap intracorporeal anastomosis. At our institution, intracorporeal anastomosis is generally selected as the standard approach, except in cases where preoperative bowel preparation cannot be performed or when the patient presents with bowel obstruction symptoms, such as dilated intestines due to impaired passage. A Powered Echelon Flex (Ethicon) 60-mm gold cartridge was inserted so that the mesenteric counterparts of the anastomotic intestine were aligned, and the anastomotic opening was created. The instrument insertion opening was closed with a 3-0 Loc (Medtronic, Minneapolis, MN, USA) with an Albert-Lembert suture (**Fig. 5**). The elevated jejunum was located directly at the superior mesenteric vein (SMV) dorsal to the anastomotic site, with an approximate distance of 0 cm between them. The operative time was 7 hours and 16 minutes, and the blood loss was 203 mL.

### Pathological findings

**Fig. 6:**
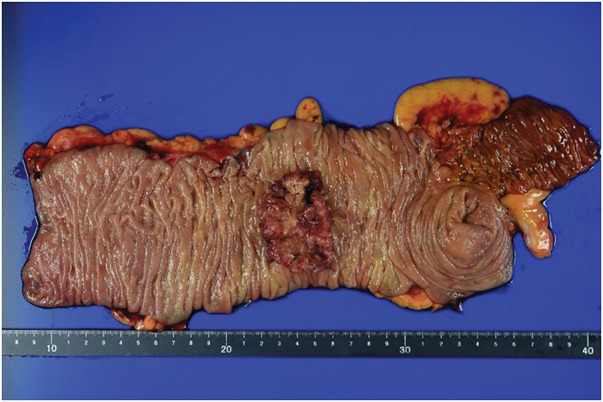
Surgical specimen macroscopic findings of the resected specimen showed a 57 × 37 mm Type 2 tumor in the ascending colon with a proximal margin of 235 mm and a distal margin of 120 mm.

Grossly, a Type 2 tumor measuring 57 × 37 mm was identified in the ascending colon. Histologically, the tumor was classified as adenocarcinoma, Type 2, 57 × 37 mm, tub2, pT3, INFb, Ly0, V1b, Pn0, pPM0 (235 mm), pDM0 (120 mm), pN0, cM0, pStage IIa, Cur A (UICC 9th edition) (**Fig. 6**).

### Postoperative course

The patient was discharged without complications on POD 8. He received postoperative adjuvant chemotherapy (tegafur-uracil+ calcium folinate) for 6 months. He has been alive for one year after the surgery, with no evidence of cancer recurrence.

## DISCUSSION

In the present case, we performed intracorporeal anastomosis during laparoscopic right hemicolectomy in a patient with a history of total gastrectomy with Roux-en-Y reconstruction. In such patients, performing extracorporeal anastomosis requires extensive adhesiolysis between the reconstructed elevated jejunum and the transverse mesocolon, as well as mobilization of the transverse colon to the left of the elevated jejunum. During this process, injury to the mesenteric vessels of the reconstructed jejunum may lead to ischemia, potentially necessitating re-anastomosis of the esophagojejunostomy. In contrast, by selecting intracorporeal anastomosis, we were able to minimize mobilization of the transverse colon to the right of the elevated jejunum, thereby avoiding injury to the reconstructed jejunum and enabling a safe anastomosis. This case highlights that intracorporeal anastomosis may represent a safe and effective surgical option in similar clinical scenarios.

In recent years, the use of intracorporeal anastomosis has been gradually increasing.^1)^ One of the surgical advantages of intracorporeal anastomosis is that it minimizes bowel dissection and mobilization.^2–14)^ Its utility has also been reported in obese patients,^15)^ and the decreased risk of bleeding associated with bowel traction or elevation is considered an additional advantage. Furthermore, because bowel dissection and mobilization are performed entirely within the abdominal cavity, the surgical specimen can be extracted through an incision at any site on the abdominal wall. Notably, intracorporeal anastomosis eliminates the need for bowel traction, thereby allowing for a smaller extraction incision compared with extracorporeal anastomosis. This approach has also been reported to reduce the incidence of postoperative incisional hernia.^16)^ Several systematic reviews and meta-analyses have evaluated the short-term outcomes of intracorporeal anastomosis and have suggested that it is associated with earlier recovery of bowel function, a lower incidence of postoperative complications, and a reduced length of hospital stay.^12–15)^ When performing intracorporeal anastomosis, several factors may contribute to improved outcomes, including reduced traction and manipulation of the bowel and mesentery, decreased postoperative pain, and a subsequent reduction in the use of analgesics.^1)^ On the contrary, concerns remain regarding the long-term oncological safety of intracorporeal anastomosis. It has been suggested that performing an anastomosis within the abdominal cavity may increase the risk of intraperitoneal dissemination of tumor cells present within the bowel lumen. Ikehara et al.^17)^ reported that cytological examination of linear staplers used for anastomosis detected cancer cells in 20% of cases, suggesting a potential risk of tumor cell dissemination. These findings highlight the importance of implementing appropriate measures to prevent intraperitoneal tumor cell dissemination when performing intracorporeal anastomosis. In our case, preoperative management included both chemical and mechanical bowel preparation. During surgery, gauze was placed under the intestinal segment to prevent contamination by minimizing the risk of intraoperative spillage of bowel contents. In addition, a retrospective study with a limited sample size reported no significant differences between the intracorporeal and extracorporeal anastomosis groups for local recurrence rates, 3-year recurrence-free survival (RFS), or overall survival (OS).^18)^ However, sufficient evidence regarding the long-term oncological safety of intracorporeal anastomosis remains insufficient, and further prospective studies are warranted. Accordingly, when performed with appropriate precautions to minimize the risk of tumor cell dissemination, intracorporeal anastomosis may be considered a reasonable and acceptable option in current clinical practice.

A PubMed search using the keywords “gastrectomy,” “ascending colon cancer,” and “transverse colon cancer” for the period from January 1991 to June 2024 identified three relevant case reports, excluding conference abstracts.^19–21)^ Ono et al.^20)^ reported a case of laparoscopic right hemicolectomy performed for transverse colon cancer in a patient with a history of gastrectomy and Billroth II reconstruction. Because the tumor was located in the mid-transverse colon, lymphadenectomy at the root of the middle colic artery and distal colonic transection were required on the left side of the elevated jejunum, necessitating adhesiolysis between the transverse mesocolon and the elevated jejunum. To preserve the blood supply to the elevated jejunum, meticulous dissection along the correct anatomical plane was essential, requiring advanced surgical expertise. Preoperative CT imaging is useful for evaluating the anatomical relationship of the bowel to address this technical challenge. In this case, a functional end-to-end anastomosis was performed extracorporeally. Although laparoscopic adhesiolysis has been reported to be safe, prompt conversion to open surgery is warranted if dense adhesions pose a risk of injury to the elevated jejunum or its mesentery. In the report by Funatsuya et al.,^21)^ intracorporeal anastomosis was selected for a case of transverse colon cancer located close to the right side of the elevated jejunum. The distal transection was performed on the right side of the elevated jejunum, resulting in a relatively short distal resection margin of approximately 5 cm. Consequently, a surgical approach was chosen that partially compromised oncological curability. In elderly patients or those with significant comorbidities, surgical procedures are generally selected based on a balance between oncological curability and the avoidance of complications. Intracorporeal anastomosis may contribute to achieving this balance. Based on the present findings, even in cases where extensive adhesions are anticipated following total gastrectomy, intracorporeal anastomosis appears to be a valuable technique that can reduce the risk of bowel injury while maintaining both oncological curability and surgical safety. It may also be particularly beneficial in cases where surgical safety must be prioritized.

## CONCLUSIONS

In cases of ascending colon cancer following total gastrectomy with Roux-en-Y reconstruction, where extensive adhesions are anticipated, intracorporeal anastomosis, which minimizes the extent of dissection and mobilization, could be considered as a treatment option. This case report demonstrates the effectiveness of intracorporeal anastomosis for colorectal cancer with a history of abdominal surgery.

## DECLARATIONS

### Concent for publication

Written informed consent was obtained from the patient for publication of this case report and any accompanying images.
